# Role of genomic DNA methylation in detection of cytologic and histologic abnormalities in high risk HPV-infected women

**DOI:** 10.1371/journal.pone.0210289

**Published:** 2019-01-04

**Authors:** Wiyada Dankai, Surapan Khunamornpong, Sumalee Siriaunkgul, Aungsumalee Soongkhaw, Arphawan Janpanao, Utaiwan Utaipat, Nakarin Kitkumthorn, Apiwat Mutirangura, Jatupol Srisomboon, Suree Lekawanvijit

**Affiliations:** 1 Gynecologic Cancer Research Cluster, Faculty of Medicine, Chiang Mai University, Chiang Mai, Thailand; 2 Department of Pathology, Faculty of Medicine, Chiang Mai University, Chiang Mai, Thailand; 3 Research Institute for Health Sciences (RIHES), Chiang Mai University, Chiang Mai, Thailand; 4 Department of Oral Biology, Faculty of Dentistry, Mahidol University, Bangkok, Thailand; 5 Center of Excellence in Molecular Genetics of Cancer and Human Diseases, Department of Anatomy, Faculty of Medicine, Chulalongkorn University, Bangkok, Thailand; 6 Department of Gynecology, Faculty of Medicine, Chiang Mai University, Chiang Mai, Thailand; Istituto Nazionale Tumori IRCCS Fondazione Pascale, ITALY

## Abstract

Cervical cancer is the fourth most common malignancy affecting women worldwide. The development of disease is related to high-risk human papillomavirus (hrHPV) infection. Cytology has been the most recommended triage for primary cervical (pre)cancer screening despite relatively low sensitivity. Recently, genomic DNA methylation has been proposed as an additional marker to increase sensitivity for detecting cervical precancerous lesion. This study aimed to evaluate the performance of methylation status of three tumor suppressor genes (*CADM1*, *FAM19A4*, and *MAL*) and HPV genotyping in detection of cytologic and histologic abnormalities in cervical cancer screening. Two hundred and sixty samples with available frozen cell pellets including 70 randomly selected cases of negative for intraepithelial lesion or malignancy (NILM)&HPV-negative, 70 randomly selected cases of NILM&HPV-positive, and 120 cytologic abnormalities & HPV-positive from a population-based cervical cancer screening program (n = 7,604) were investigated for the DNA methylation pattern of *CADM1*, *FAM19A4*, and *MAL*. Of 120 cytologic abnormalities & HPV-positive cases, there were 115 available histologic results. HPV52 and HPV58 were most commonly found in histologic HSIL+. The methylation levels of *CADM1*, *FAM19A4*, and *MAL* were elevated with the severity of cytologic abnormality which significantly increased by 3.37, 6.65 and 2 folds, respectively, in cytologic HSIL comparing with NILM. A significant increase in methylation levels of these three genes was also observed in histologic HSIL+ compared with negative histology but only *CADM1* showed a significant higher methylation level than histologic LSIL. Using the ROC curve analysis, DNA methylation levels of *FAM19A4* performed best in differentiating high-grade cytology (ASC-H+ from NILM/ASC-US/LSIL), followed by *CADM1* and *MAL*. Whilst the *CADM1* methylation performed best in distinguishing histologic HSIL+ from negative/LSIL with an area under the ROC curve of 0.684, followed by *MAL* (0.663) and *FAM19A4* (0.642). Interestingly, after combining high DNA methylation levels to HPV16/18 genotypes, rates of histologic HSIL+ detection were substantially increased from 25% to 79.55% for *CADM1*, 77.27% for *FAM19A4*, and 72.73% for *MAL*, respectively. The rate further increased up to 95.45% when at least one of three genes had a high methylation level. This suggests a possible role of genomic DNA methylation, especially *CADM1*, in detecting histologic HSIL+ lesions in combination with hrHPV testing.

## Introduction

Cervical cancer is the fourth most common malignancy affecting women worldwide [1]. In 2012, the global numbers of 528,000 new cervical cancer cases and 266,000 deaths were estimated by GLOBOCAN [1]. Cervical cancer is related with human papillomavirus (HPV) especially high-risk HPV (hrHPV), with HPV16 being the most common genotype (60%) [2]. However, HPV infection only is not sufficient to induce carcinogenesis. The genetic and epigenetic changes in the host and/or viral genome have been demonstrated to be associated with the progression to invasive cancerous lesion [3].

Cytology is still used for primary cervical cancer screening despite relatively low sensitivity (51% from a meta-analysis) [4]. Over the past decade, hrHPV DNA testing has been recommended in the cervical cancer screening program. With a higher sensitivity but a lower specificity than cytology [5], hrHPV test is usually used as a co-test with cytology. Recently, the aberrant promoter methylation of tumor suppressor genes has been shown to contribute to cervical carcinogenesis [6–10] in hrHPV-positive women. Several DNA methylation markers involving in carcinogenesis are being developed as tools for predicting progressive lesions. The promoter hypermethylation-mediated silencing of tumor suppressor genes such as cell adhesion molecule 1 (*CADM1*) [7, 8], T-lymphocyte maturation associated protein (*MAL*) [9], and family with sequence similarity 19 member A4, C-C motif chemokine like (*FAM19A4*) genes [10] have been reported to play a role in cervical carcinogenesis.

*CADM1*, originally *TSLC1*, encodes a transmembrane protein of the immunoglobulin superfamily which involves in epithelial cell adhesion [11]. The functional involvement in cervical carcinogenesis of *CADM1* has been demonstrated by Steenbergen and colleagues in 2004 [7]. Methylation-mediated silencing of the *CADM1* has been reported in cervical carcinoma cell line [7, 12], advanced stage of cervical intraepithelial neoplasia (CIN) lesion and cervical squamous cell carcinoma (SCC) lesion in hrHPV-positive women [7, 8]. Moreover, Overmeer and colleagues (2008) reported that the dense methylation (≥ 2 methylated regions) of *CADM1* was stronger associated with CINIII+ compared with CINI, similar to SCC lesion compared with adenocarcinomas, in hrHPV-positive women [8].

*MAL* encodes the T-lymphocyte maturation-associated protein which involves in membrane trafficking processes in epithelial cells. Suppression of *MAL* expression by promoter methylation is associated with cervical carcinogenesis [9]. The promoter hypermethylation of *MAL* was frequently detected in hrHPV-positive samples with high-grade cervical lesions, especially CIN III, SCC and adenocarcinoma [9]. In addition, combined *CADM1* and *MAL* promoter methylation has been proposed as the bi-marker panel for cervical screening in hrHPV positive women [13–16].

*FAM19A4* is a member of the TAFA family of five highly homologous genes that encode small secreted proteins. These proteins are related to the MIP-1α protein which serves as immuno-regulators and chemokines [17]. Genome-wide methylation study of cervical cancer has been reported that the promoter hypermethylation of *FAM19A4* was frequently detected in all cervical carcinoma [18]. Moreover, methylation of *FAM19A4* has been proposed as an alternative biomarker for early detection of cervical cancer, particularly high-grade squamous intraepithelial lesions (HSIL) [10, 19], as well as a new triage tool for self-sample collection [20] in hrHPV-positive women.

A few studies suggested that *CADM1*, *FAM19A4* and *MAL* methylation markers were potentially high-performance markers for cervical screening [21–23]. Based on these findings, we hypothesized that the addition of these methylation markers in our cervical screening program will be a valuable triage for detecting cervical cancer in HPV-infected women in Northern Thailand.

Several studies on the detection of cervical cancer using DNA methylation marker usually focus on the two well-known common hrHPV genotypes, HPV16 or HPV18 [19, 24]. However, the prevalence and genotypic distribution of HPV vary across geographic regions. HPV52 and HPV58 were highly prevalence in Asia [25, 26], particularly in Northern Thailand [27]. This study therefore aimed to determine the relationship between methylation status of 3 tumor suppressor genes (*CADM1*, *FAM19A4*, and *MAL*) using methylation-specific polymerase chain reaction (MSP) technique and abnormal cytology/histology cervical lesions in HPV-infected women in Northern Thailand.

## Materials and methods

### Study population

This study was approved by the institutional ethics committee of the Faculty of Medicine, Chiang Mai University (study code: HOS-2559-04347). The study population was part of population-based cervical cancer screening in Northern Thailand scheduled by the Ministry of Health during June 2014 to December 2015 which was approved by the institutional ethics committee of the Faculty of Medicine, Chiang Mai University (study code: PAT-11-02-07A-14-X) (n = 7,604). Given that this was a retrospective study that used registry data gathered by the laboratory of the Pathology Department, Faculty of Medicine, the ethics committee waived the requirement for informed consent. Data were anonymously analyzed.

Cervical samples were collected from women aged 25 to 60 years. Exclusion criteria were cervical specimens from patients with conditions as follow: pregnancy, previous hysterectomy, or previous history of abnormal cervical epithelial lesions. All cervical specimens were collected for 1) Pap smear 2) HPV DNA test and 3) cell pellet collection. Samples for HPV test and cell pellet collection were kept at 4°C and immediately transferred to the pathology laboratory. The process of cell pellet collection was done within 4 hours and the cell pellets were stored at -80°C until methylation analysis. HPV test was performed on the following day.

All abnormal cytology specimens were retrieved for this study. Samples with cytologically negative for intraepithelial lesions or malignancy (NILM) were also randomly selected from HPV-negative and HPV-positive women (70 samples each). A total number of 260 samples were finally included in this study and were divided into 6 groups (Table 1). A frozen cell pellet from all sample were subjected to evaluate the DNA methylation status of *CADM1*, *FAM19A4*, and *MAL* genes. All women with cytologic abnormalities were followed up for histologic diagnosis by colposcopy directed biopsy (CDB) and/or loop electrosurgical excision procedure (LEEP). Of 120 abnormal cytologic samples [Atypical squamous cells of undetermined significance or worse (ASC-US+)], there were 115 samples with available histologic results.

**Table 1:** Study population groups characterized by cytology and HPV status.

Groups	Number	Percentage
Group1. NILM&HPV-negative	70	26.92
Group2. NILM&hrHPV-positive	70	26.92
Group3. ASC-US&hrHPV-positive	48	18.46
Group4. LSIL,&hrHPV-positive	34	13.07
Group5. ASC-H&hrHPV-positive	22	8.46
Group6. HSIL&hrHPV-positive	16	6.15
**Total**	**260**	**(100)**

ASC-H; Atypical squamous cells, cannot exclude HSIL, ASC-US; Atypical squamous cells of undetermined significance, HSIL; High-grade squamous intraepithelial lesion, LSIL; Low-grade squamous intraepithelial lesion, NILM; Negative for intraepithelial lesions or malignancy

### HPV detection and genotyping

All samples were subjected to detect hrHPV (14 genotypes) DNA using the Cobas 4800 HPV test (Roche Molecular Systems, USA), a fully-automated platform based on real-time PCR technique. Samples with hrHPV positivity were subsequently genotyped by using the Linear Array (LA) HPV genotyping test (Roche Molecular Systems, Pleasanton, CA).

### Cell culture

The Ca Ski human cervical cancer cell line (Ca Ski, American Type Culture Collection) harboring 600 copies of HPV16 per cell was used as a positive control for methylation analysis. 8.5 x 10^5^ Ca Ski cells were cultured in T-25 flask (Nunc, Shanghai) with 6 ml of DMEM (Gibco, life technologies) supplemented with 10% FBS (Biochrom GmbH, Germany). Cells were grown at 37°C in 5% CO_2_ incubator until reaching 90% confluence before DNA extraction.

### DNA isolation and sodium bisulfite modification

Genomic DNA was extracted from frozen cervical cell pellets and Ca Ski cells using the Qiamp DNA extraction kit (QIAGEN GmbH, Germany). DNA quality and quantity were determined by NanoDrop spectrophotometer (Nanodrop 2000, Thermo Fisher Scientific, Waltham, MA, USA). The extracted DNA was subjected to bisulfite treatment using the EZ DNA Methylation-Gold kit (Zymo Research, CA, USA). Briefly, 750 ng DNA was denatured before bisulfite conversion in one step using the following condition: 98°C for 10 minutes and 64°C for 2.5 hours. Then the modified DNA was cleaned up and desulphonated before being stored at -20°C until used.

### Methylation-specific polymerase chain reaction (MSP)

MSP was performed to evaluate the methylation status of 3 markers (*CADM1*, *FAM19A4* and *MAL*). Bisulfite treated DNA (75 ng) was used for each MSP reaction which contained two primer pairs (methylation and unmethylation) for one specific gene (Table 2).

**Table 2:** The primer sets for methylation specific PCR.

Primer name	Sequence	Product size (bp)	Tm (°C)	Reference
CADM1 MSP-M1 (F)	GAA AAT TTT AGA ATT CGA TTT TAC G	114	58	Overmeer, et al., 2008
CADM1 MSP-M1 (R)	AAA ATA CAT ACG TAC TTT ACA CG			
CADM1 MSP-U1 (F)	GAA AAT TTT AGA ATT TGA TTT TAT G	117	57	
CADM1 MSP-U1 (R)	AAA AAA ATA CAT ACA TAC TTT ACA CA			
MAL MSP-M (F)	TTC GGG TTT TTT TGT TTT TAA TTC	139	56	Lind et al., 2008
MAL MSP-M (R)	GAA AAC CAT AAC GAC GTA CTA ACG T			
MAL MSP-U (F)	TTT TGG GTT TTT TTG TTT TTA ATT T	142	56	
MAL MSP-U (R)	ACA AAA ACC ATA ACA ACA TAC TAA CAT C			
FAM19A4 MSP-M (F)	TAG CGC GTT TCG CGG CGG	75	57	
FAM19A4 MSP-M (R)	CGC AAT ACG AAA CCG AAC CCA AC			
FAM19A4 MSP-U (F)	GTG TGT TTT GTG GTG GGT TTG G	119	53	
FAM19A4 MSP-U (R)	CCC ACA ACC ACA CAC ACA ATC A			

M; methylation specific primer, U; unmethylation specific primer, (F); forward primer, (R); reverse primer

The PCR reaction mixture comprised 1 μl (75 ng) of bisulfite treated DNA, 1X PCR reaction buffer, 0.2 mM dNTP mixture, 1.5 mM MgCl_2_, 0.2 μM each forward and reverse primer, and 1 unit of Platinum *Taq* DNA Polymerase (Invitrogen, Brazil) in a total reaction volume of 20 μl. The PCR amplification was performed with an initial denaturing step at 94°C for 7 minutes, then 40 cycles of the following conditions: 94°C for 30 seconds, 58°C for 30 seconds and 72°C for 45 seconds. The final extension was at 72°C for 7 minutes.

The PCR products were separated on 2% agarose electrophoresis gel and bands were visualized by Redsafe Nucleic Acid Staining Solution (iNtRON Biotechnology, Inc.). The band intensity was measured using the gel documentation system (ChemiDoc Touch Imaging System, Bio-rad). The percentage of methylation was calculated using the following formula;

$$\%\mathrm{methylation}=\left(\mathrm{Band}\;{\mathrm{intensity}}_{\mathrm{methylation}}/\mathrm{Band}\;{\mathrm{intensity}}_{\mathrm{methylation}+\mathrm{unmethylation}}\right)\;x\;100$$

Where;

Band intensity_methylation_ = Intensity of methylation band

Band intensity_methylation + unmethylation_ = Intensity of methylation band plus unmethylation band

### Statistical analysis

The differences of HPV genotypes and DNA methylation levels among groups (categorized by cytology&HPV status and histologic grades) were analyzed using ANOVA analysis. The correlation between DNA methylation levels and cytologic or histologic abnormalities was analyzed using Spearman’s rho.

The performance of each methylation marker to distinguish cytologic abnormalities [Atypical squamous cells cannot exclude HSIL or worse (ASC-H+) vs. NILM/ASC-US/ low-grade squamous intraepithelial lesion (LSIL) and ASC-US+ vs. NILM] and histologic abnormalities (histologic HSIL+ vs. negative/LSIL and histologic LSIL+ vs. negative) was evaluated by the receiver operating characteristic (ROC) curve analysis using the best cutoff values with the maximum sum of sensitivity and specificity. In addition, the multiple logistic regression analysis was used to determine the correlation between histologic HSIL+ and DNA methylation levels and HPV genotypes. The HPV status was classified into 4 groups based on the IARC HPV classification and highly prevalent genotypes found in Northern Thailand as the following; 1) low-risk HPVs type (6, 11, 40, 42, 54, 61, 70, 72, 81 and CP6108), 2) probable hrHPVs type (26, 53 and 66), 3) carcinogenic group (HPV type 31, 33, 35, 39, 45, 51, 56, 59, 68, 73 and 82), and 4) most common HPV genotypes found in Northern Thailand (HPV type 16, 18, 52, and 58) [27]. P values < 0.05 were considered statistically significant. Statistical analyses were performed using SPSS version 17.

## Results

### General characteristics of the study population

Among 7,604 women aged 25 to 60 years were enrolled in the cervical cancer screening program, 7,088 (93.21%) samples were negative both cytology and hrHPV tests, 44 (0.58%) samples were only positive for cytology, 312 (4.1%) were only positive for hrHPV test, and 160 (2.1%) samples were positive for both tests. Of these 160 samples, there were 120 adequate cell pellet samples available for this methylation study and 115 with available histologic results.

The histologic results included 39 (33.91%) negative, 32 (27.83%) LSIL, 42 (36.52%) HSIL, and 2 (1.74%) SCC samples. The relationship between cytologic results and histologic diagnoses was shown in Table 3.

**Table 3:** The relationship between cytologic results and histologic diagnoses.

		Histology			
Cytology	Negative (n = 39)	LSIL (n = 32)	HSIL (n = 42)	SCC (n = 2)	Total
ASC-US	23 (51.1%)	13 (28.9%)	9 (20%)	-	45 (100%)
LSIL	12 (36.4%)	15 (45.4%)	6 (18.2%)	-	33 (100%)
ASC-H	3 (14.3%)	2 (9.5%)	16 (76.2%)	-	21 (100%)
HSIL	1 (6.3%)	2 (12.5%)	11 (68.7%)	2 (12.5%)	16 (100%)

ASC-H; Atypical squamous cells, cannot exclude HSIL, ASC-US; Atypical squamous cells of undetermined significance, HSIL; High-grade squamous intraepithelial lesion, LSIL; Low-grade squamous intraepithelial lesion, SCC; Squamous cell carcinoma

### HPV genotyping

**Fig 1:**
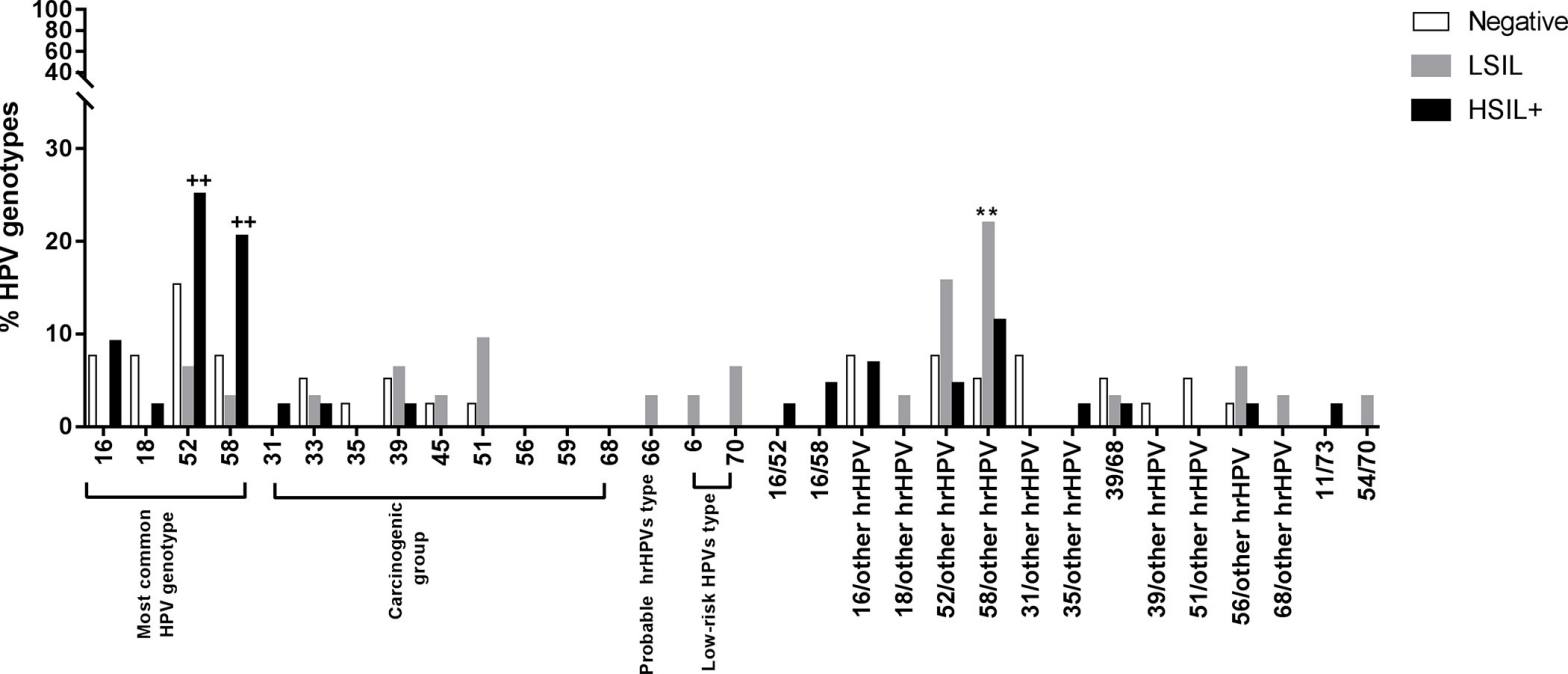
The prevalence of HPV genotypes in the difference histologic grades (ANOVA with turkey’s multiple comparison post hoc test: ** p < 0.01 vs. negative histology and ++ p < 0.01 vs. histologic LSIL).

Of 115 samples with available histology and genotyping results, there were 58 (50.43%) single infections, 50 (43.47%) multiple infections, and 7 (6.08%) non-14hrHPV infections (Table 4). HPV52 and HPV58 were most commonly found in both single and multiple infections (Fig 1).

**Table 4:** Genotyping results of 115 women with available histology.

HPV genotype	Histology results, n = 115		
	Negative n = 39 (100%)	LSIL n = 32 (100%)	HSIL+ n = 44 (100%)
**Single infection, n = 58**			
HPV16, n = 7	3 (7.69%)	0	4 (9.09%)
HPV18, n = 4	3 (7.69%)	0	1 (2.27%)
HPV31, n = 1	0	0	1 (2.27%)
HPV33, n = 3	2 (5.12%)	0	1 (2.27%)
HPV35, n = 1	1 (2.56%)	0	0
HPV39, n = 3	2 (5.12%)	0	1 (2.27%)
HPV45, n = 2	1 (2.56%)	1 (3.12%)	0
HPV51, n = 4	1 (2.56%)	3 (9.37%)	0
HPV52, n = 19	6 (15.38%)	2 (6.25%)	11 (25%)
HPV56, n = 0	0	0	0
HPV58, n = 13	3 (7.69%)	1 (3.12%)	9 (20.45%)
HPV59, n = 0	0	0	0
HPV66, n = 1	0	1 (3.12%)	0
HPV68, n = 0	0	0	0
**Multiple infection, n = 50**			
HPV16/52, n = 1	0	0	1 (2.27%)
HPV16/58, n = 2	0	0	2 (4.54%)
HPV16/hrHPVs, n = 6	3 (7.69%)	0	3 (6.81%)
HPV18/hrHPVs, n = 1	0	1 (3.12%)	0
HPV52/hrHPVs, n = 10	2 (5.12%)	6 (18.75%)	2 (4.54%)
HPV58/hrHPVs, n = 14	2 (5.12%)	7 (21.87%)	5 (11.36%)
HPV31/hrHPVs, n = 4	3 (7.69%)	1 (3.12%)	0
HPV35/hrHPVs, n = 1	0	0	1 (2.27%)
HPV39/hrHPVs, n = 4	3 (7.69%)	1 (3.12%)	0
HPV51/hrHPVs, n = 1	1 (2.56%)	0	0
HPV56/hrHPVs, n = 4	1 (2.56%)	2 (6.25%)	1 (2.27%)
HPV66/hrHPVs, n = 1	1 (2.56%)	0	0
HPV68/hrHPVs, n = 1	0	1 (3.12%)	0
**Non-14hrHPVs infection, n = 7**			
HPV6, n = 1	0	1 (3.12%)	0
HPV11/73, n = 1	0	0	1 (2.27%)
HPV54,71, n = 1	0	1 (3.12%)	0
HPV62, n = 1	1 (2.56%)	0	0
HPV67, n = 1	0	1 (3.12%)	0
HPV70, n = 2	0	2 (6.25%)	0

HPV; human papillomavirus, hrHPV; high-risk human papillomavirus, HSIL+; High-grade squamous intraepithelial lesion or worse, LSIL; Low-grade squamous intraepithelial lesion

For single infection, HPV52 and HPV58 genotypes were significantly higher in histologic HSIL+ compared with histologic LSIL, 25% (11 of 44) vs 6.25% (2 of 32), p = 0.003 for HPV52 and 20.45% (9 of 44) vs 3.12% (1 of 32), p = 0.006 for HPV58 (Fig 1).

### The methylation levels of *CADM1*, *FAM19A4* and *MAL* were increased with the severity of cytologic and histologic abnormalities

**Fig 2:**
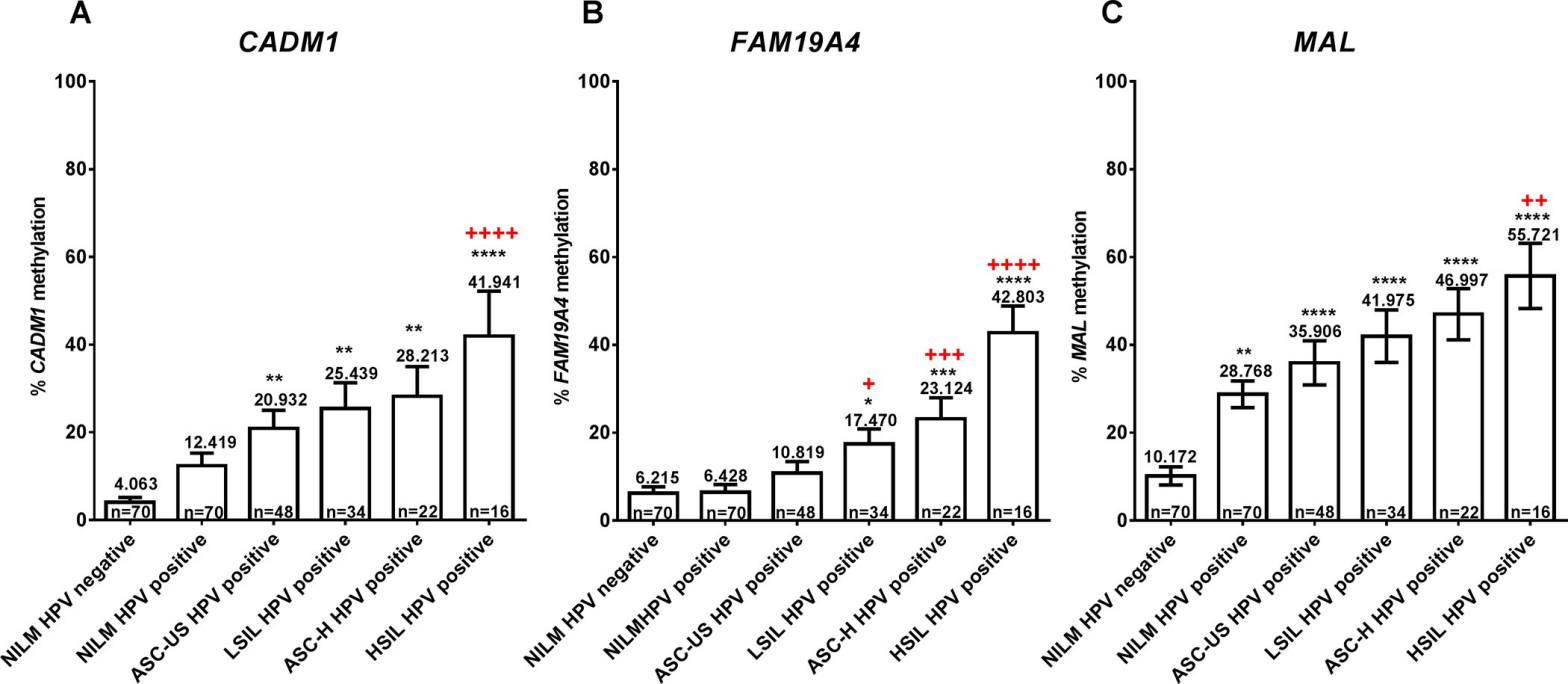
The methylation levels of three candidate genes; *CADM1* (A), *FAM19A4* (B), and *MAL* (C), in the different cytologic grades (ANOVA with turkey’s multiple comparison post hoc test * p < 0.05, ** p < 0.01, *** p < 0.001, **** p < 0.0001 vs. NILM&HPV-negative group, and + p < 0.05, ++ p < 0.01, +++ p < 0.001, ++++ p < 0.0001 vs. NILM&HPV-positive group).

Methylation levels of all candidate genes increased with the severity of cytologic abnormality in a stepwise fashion (Fig 2). The methylation levels of *MAL* gene showed a significant increase in all HPV-positive samples. Whilst the levels of *CADM1* and *FAM19A4* methylation significantly increased in cytologic ASC-US+&HPV-positive and cytologic LSIL+&HPV-positive groups, respectively, when compared with NILM&HPV-negative group (Fig 2). Among HPV-positive samples, the methylation levels of *CADM1*, *FAM19A4* and *MAL* were significantly increased by 3.37, 6.65 and 2 folds, respectively, in cytologic HSIL comparing with NILM.

**Fig 3:**
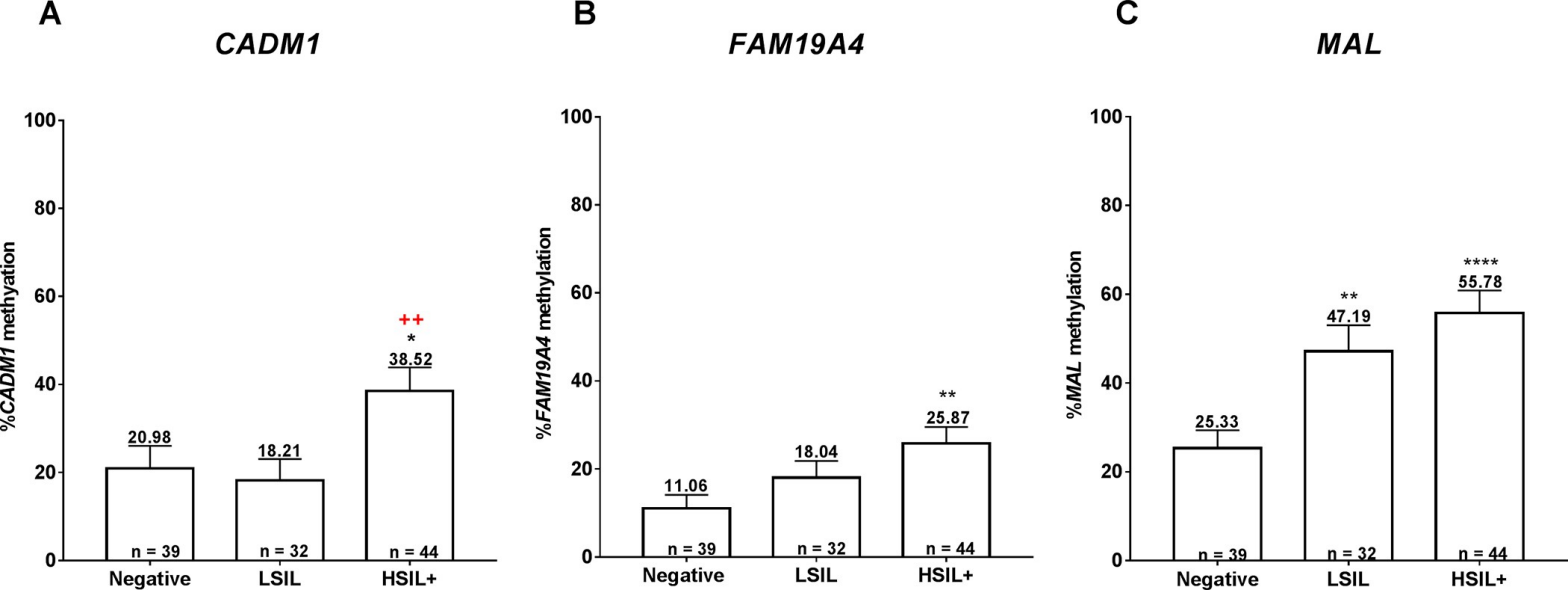
The methylation levels of three candidate genes; *CADM1* (A), *FAM19A4* (B), and *MAL* (C), in the different histologic grades (ANOVA with turkey’s multiple comparison post hoc test * p < 0.05, ** p <0.01, **** p <0.0001 vs. negative and + p < 0.05 vs. histologic LSIL).

When considering histologic abnormalities among hrHPV-positive women with cytologic ASC-US+, the methylation levels of all three genes were significantly higher in histologic HSIL+ than negative histology (Fig 3). In addition, a significant increase in *CADM1* methylation levels was observed in histologic HSIL+ compared with LSIL groups (Fig 3A) while that of *MAL* was observed in histologic LSIL+ compared with negative groups (Fig 3C).

### Correlation between methylation markers, cytologic abnormality, and histologic abnormality

Using the Spearman’s rho correlation, the methylation levels of all three candidate genes were positively correlated with cytologic grades in HPV-positive sample [r = 0.271 (*CADM1*), 0.422 (*FAM19A*), 0.258 (*MAL*); p < 0.01]. Similar positive correlations were also observed with histologic grades [Spearman’s rho correlate; r = 0.279 (*CADM1*), r = 0.295 (*FAM19A4*), r = 0.361 (*MAL*); p < 0.01].

### Performance of DNA methylation markers to distinguish cytologic abnormalities

**Fig 4:**
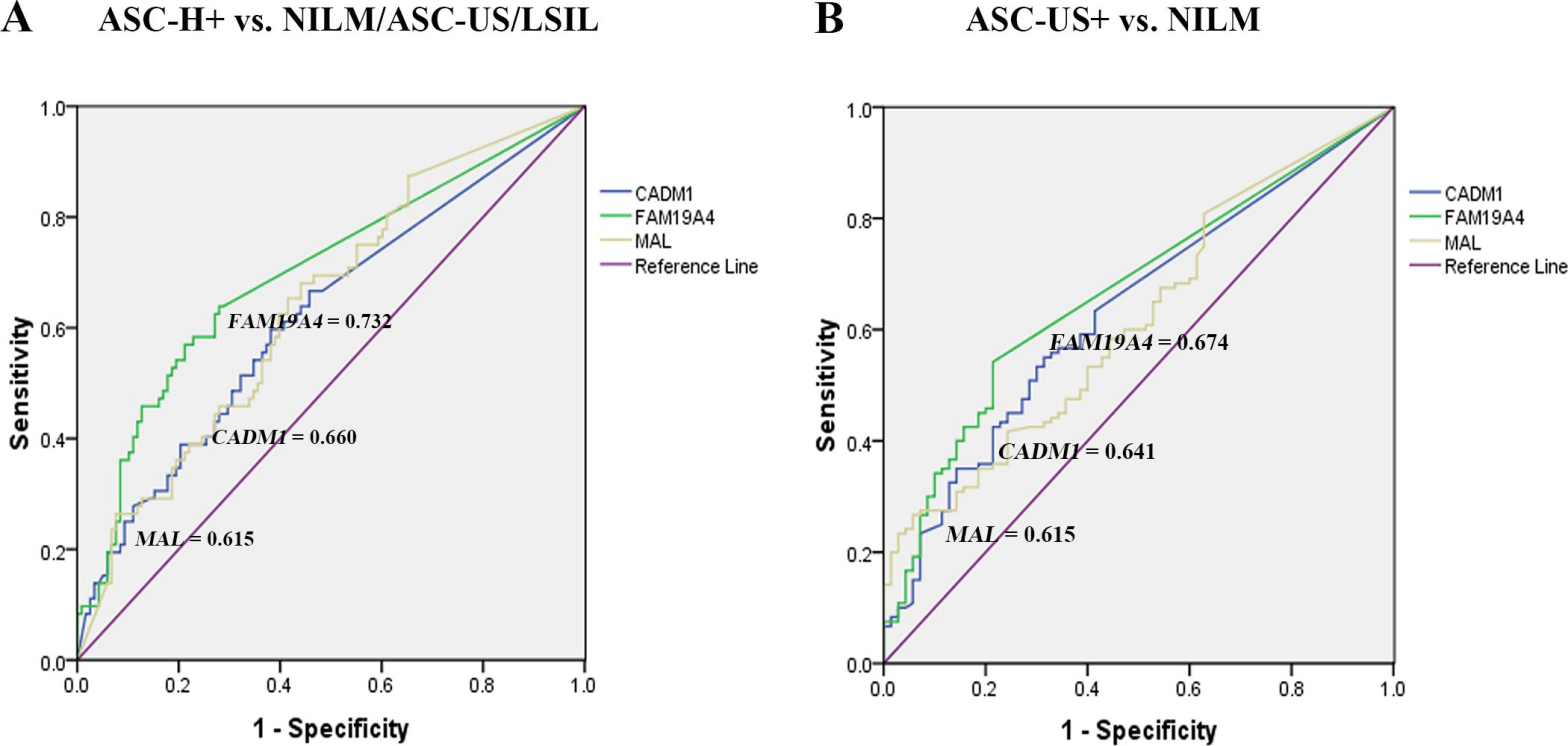
The ROC curve analysis of three methylation markers in differentiating cytologic ASC-H+ from NILM/ASC-US/LSIL (A) and cytologic ASC-US+ from NILM (B).

Using the best cutoff values with the maximum sum of sensitivity and specificity (Table 5), DNA methylation levels of *FAM19A4* performed best followed by *CADM1* and *MAL* in differentiating high-grade cytologic abnormalities ASC-H+ from NILM/ASC-US/LSIL (Fig 4A) as well as in differentiating cytologic abnormalities ASC-US+ from NILM (Fig 4B).

**Table 5:** The cutoff values of DNA methylation markers for distinguishing high-grade cytologic abnormalities (ASC-H+ vs. NILM/ASC-US/LSIL) and cytologic abnormalities (ASC-US+ vs. NILM) in HPV-positive women.

DNA methylation markers	Best cutoffs (% methylation)	Sensitivity % (95% CI)	Specificity % (95% CI)	PPV %	NPV %	Accuracy %	AUC (95% CI)
**Cytologic ASC-H+ vs. NILM/ASC-US/LSIL**							
***CADM1***	**8.197**	**73.7** **(56.9–86.6)**	**60.5** **(52.9–68.9)**	**31.82** **(26.2–38.0)**	**90.20** **(84.2–94.1)**	**63.16** **(55.9–70.0)**	**0.660** **(0.56–0.76)**
***FAM19A4***	**7.5**	**71.1** **(56.1–85.4)**	**68.4** **(60.4–75.7)**	**35.53** **(28.8–42.8)**	**90.35 (84.9–94.0)**	**68.42** **(61.3–74.9)**	**0.732** **(0.64–0.83)**
***MAL***	**34.78**	**76.32** **(59.8–88.6)**	**52.63** **(44.4–60.8)**	**28.71** **(24.0–34.0)**	**89.89 (83.1–94.1)**	**57.37** **(50.0–64.0)**	**0.651** **(0.56–0.74)**
**Cytologic ASC-US+ vs. NILM**							
***CADM1***	**0.909**	**63.33** **(54.1–72.0)**	**60** **(47.6–71.5)**	**72.38** **(65.8–78.1)**	**48.24 (40.7–55.9)**	**61.58** **(54.3–68.5)**	**0.6417** **(0.56–0.72)**
***FAM19A4***	**1.39**	**54.17** **(44.8–63.3)**	**80** **(68.7–88.6)**	**81.25** **(72.9–87.5)**	**50.00 (44.3–55.7)**	**63.16** **(55.8–70.0)**	**0.6743** **(0.6–0.75)**
***MAL***	**33.11**	**60** **(50.7–68.8)**	**52.86** **(40.6–64.9)**	**68.57** **(62.1–74.4)**	**43.53 (36.1–51.3)**	**57.37** **(50.0–64.5)**	**0.6155** **(0.54–0.7)**

AUC; Area under the ROC curve, ASC-H; Atypical squamous cells, cannot exclude HSIL, ASC-US; Atypical squamous cells of undetermined significance, CI; confidence interval, HSIL; High-grade squamous intraepithelial lesion, LSIL; Low-grade squamous intraepithelial lesion, NILM; Negative for intraepithelial lesions or malignancy, NPV; negative predictive value, PPV; positive predictive value

### Performance of DNA methylation markers to distinguish histologic abnormalities

**Fig 5:**
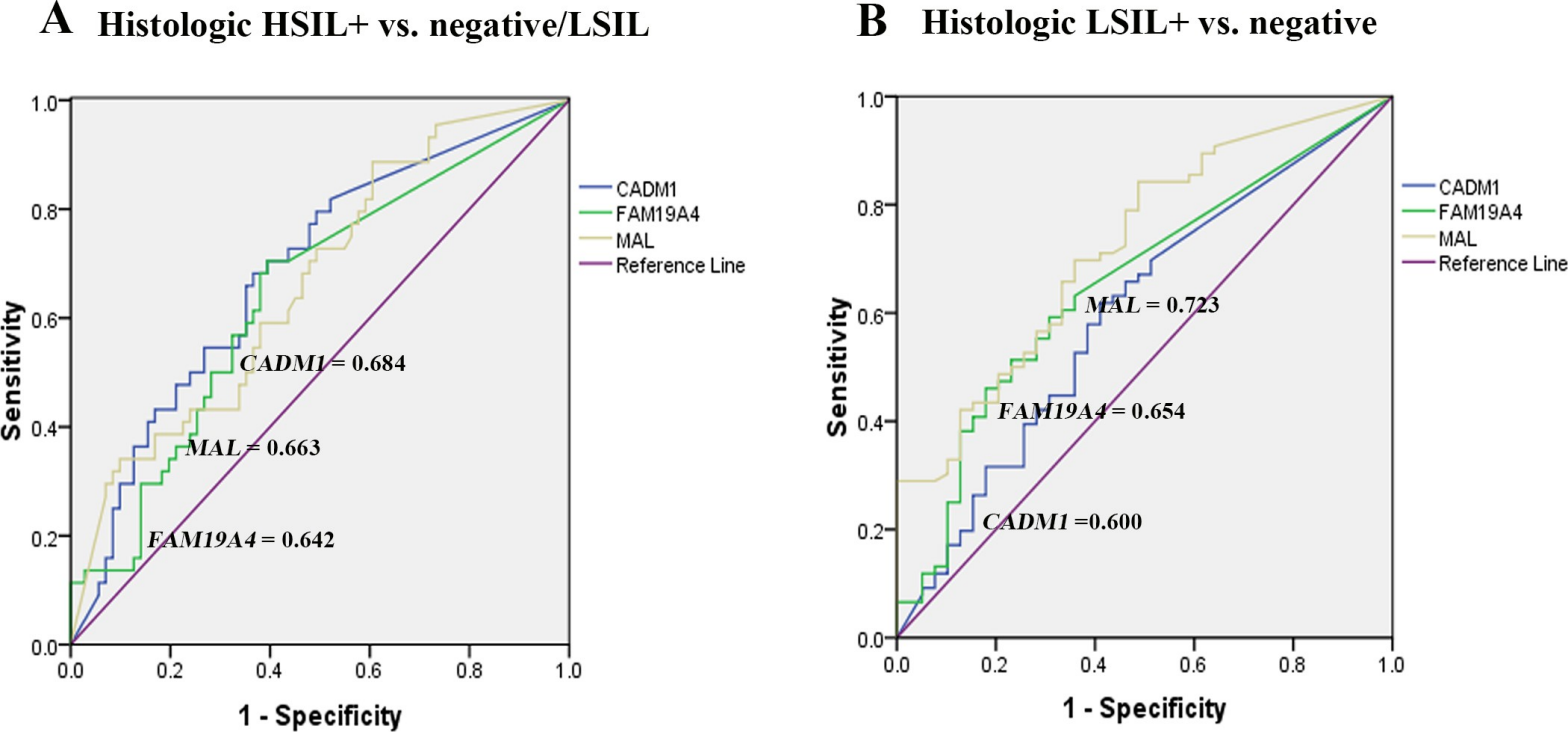
The ROC curve analysis of three methylation markers in differentiating histologic HSIL+ from negative/LSIL (A), and histologic LSIL+ from negative histology (B).

The best cutoff values for distinguishing between high-grade and non high-grade histologic abnormalities (histologic HSIL+ vs. negative/LSIL), and between histologic abnormalities and negative histology (histologic LSIL+ vs. negative) using ROC analysis were shown in Table 6. The *CADM1* methylation performed best in distinguishing high-grade histologic abnormality with an area under the ROC curve (AUC) of 0.684 followed by *MAL* (0.663) and *FAM19A4* (0.642) (Fig 5A). Despite a higher AUC of *CADM1* than *FAM19A4*, there were comparable sensitivity, in the specificity, positive predictive value (PPV), negative predictive value (NPV) and accuracy of DNA methylation of these two genes in detecting histologic HSIL+. Whereas the *MAL* methylation showed the highest AUC (0.723) in differentiating abnormal from normal histology, followed by *FAM19A4* (0.654) and *CADM1* (0.600) (Fig 5B).

**Table 6:** The cutoff values of DNA methylation marker for distinguishing high-grade histologic abnormalities (histologic HSIL+ vs. negative/LSIL) and histologic abnormalities (histologic LSIL+ vs. negative histology) in HPV-positive women.

DNA methylation markers	Best cutoffs (% methylation)	Sensitivity % (95% CI)	Specificity % (95% CI)	PPV %	NPV %	Accuracy %	AUC (95% CI)
**Histologic HSIL+ vs. negative/LSIL**							
***CADM1***	**9.009**	**70.45** **(54.8–83.3)**	**60.56** **(48.3–72.0)**	**52.54** **(44.0–61.0)**	**76.79** **(66.9–84.4)**	**64.35** **(54.9–73.6)**	**0.684** **(0.58–0.78)**
***FAM19A4***	**6.69**	**68.18** **(52.4–81.3)**	**60.56** **(48.3–72.0)**	**51.72** **(43.0–60.4)**	**75.44 (65.7–83.1)**	**63.48 (54.0–72.3)**	**0.642** **(0.54–0.75)**
***MAL***	**34.3**	**72.73** **(57.2–85.0)**	**50.7** **(38.6–62.8)**	**47.76** **(40.5–55.2)**	**75** **(63.8–83.7)**	**59.13 (49.6–68.2)**	**0.663** **(0.56–0.76)**
***CADM1/*** ***FAM19A4***	**positive**	**86.36** **(72.6–94.8)**	**40.85** **(29.3–53.2)**	**47.50** **(41.9–53.2)**	**82.86** **(68.6–91.5)**	**58.26** **(48.7–67.4)**	**0.636** **(0.53–0.74)**
***CADM1/*** ***MAL***	**positive**	**88.60** **(75.4–96.2)**	**33.80** **(23.0–46.0)**	**45.35** **(40.5–50.3)**	**82.76** **(66.4–92.1)**	**54.78 (45.2–64.1)**	**0.612** **(0.51–0.72)**
***FAM19A4/*** ***MAL***	**positive**	**79.55** **(64.70–90.2)**	**30.99** **(20.5–43.1)**	**41.67** **(36.5–47.0)**	**70.97** **(55.4–82.8)**	**49.57** **(40.1–59.0)**	**0.553** **(0.45–0.66)**
***CADM1/*** ***FAM19A4/*** ***MAL***	**positive**	**88.64** **(75.4–96.2)**	**22.54** **(13.5–34.0)**	**41.49** **(37.6–45.5)**	**76.19** **(55.8–89.0)**	**47.83** **(38.4–57.3)**	**0.556** **(0.45–0.66)**
**Histologic LSIL+ vs. negative histology**							
***CADM1***	**4.36**	**65.79** **(54.0–76.3)**	**53.85** **(37.2–69.9)**	**73.53** **(65.6–80.2)**	**44.68 (34.5–55.3)**	**61.74 (52.2–70.6)**	**0.600** **(0.49–0.71)**
***FAM19A4***	**1.39**	**63.16** **(51.3–73.9)**	**64.1** **(47.2–78.8)**	**77.42** **(68.5–84.4)**	**47.17 (38.0–56.5)**	**63.48 (54.0–72.3)**	**0.654** **(0.55–0.76)**
***MAL***	**34.3**	**69.74** **(58.1–79.8)**	**64.1** **(47.2–78.8)**	**79.10** **(70.8–85.5)**	**52.08 (41.8–62.2)**	**67.83 (58.5–76.2)**	**0.723** **(0.63–0.82)**
***CADM1/*** ***FAM19A4***	**positive**	**82.89** **(72.5–90.6)**	**38.46** **(23.4–55.4)**	**72.41** **(66.8–77.4)**	**53.57** **(37.9–68.5)**	**67.83** **(58.5–76.2)**	**0.607** **(0.49–0.72)**
***CADM1/*** ***MAL***	**positive**	**85.53** **(75.6–92.5)**	**41.03** **(25.6–57.9)**	**73.86** **(68.2–78.8)**	**59.26** **(42.8–73.8)**	**70.43** **(61.2–78.6)**	**0.633** **(0.52–0.75)**
***FAM19A4/*** ***MAL***	**positive**	**85.53** **(75.6–92.5)**	**43.59** **(27.8–60.4)**	**74.71** **(68.8–79.8)**	**60.71** **(44.6–74.8)**	**71.30** **(62.1–79.4)**	**0.646** **(0.53–0.76)**
***CADM1/*** ***FAM19A4/*** ***MAL***	**positive**	**90.79** **(81.9–96.2)**	**30.77** **(17.0–47.6)**	**71.87** **(67.2–76.1)**	**63.16** **(42.3–80.0)**	**70.43** **(61.2–78.6)**	**0.608** **(0.49–0.72)**

AUC; Area under the ROC curve CI; confidence interval, HSIL; High-grade squamous intraepithelial lesion, LSIL; Low-grade squamous intraepithelial lesion, NPV; negative predictive value, PPV; positive predictive value

When combining two or three positive methylation markers (higher than the best cutoffs) to detect either histologic HSIL+ or abnormal histology, the AUCs did not increase while the sensitivities increased and the specificities decreased, compared with single methylation markers in isolation (Table 6).

### Combining methylation markers to hrHPV genotypes improving detection of HSIL+ histology

When considering 44 cases with histologic HSIL+ from all 115 available histologic results, HPV16/18 genotypes were accounted for 25% (11 of 44) while HPV16/18/52/58 genotypes were 86.63% (38 of 44) (Table 7). We then combined the methylation markers to HPV genotyping by dividing cases into methylation ‘negative’ and ‘positive’ groups using the cutoff for distinguishing histologic HSIL+ from negative/LSIL (Table 6). Methylation positive was defined as a methylation level higher than the cutoff.

**Table 7:** Combining methylation markers to HPV genotyping in detecting histologic HSIL+ lesions.

Test	Number of cases (n = 115)	Cases with histologic HSIL+, n = 44 (%)	Ratio in detection histologic HSIL+
HPV16/18	21	11 (25%)	0.52
HPV16/18 or ***CADM1*** positive	69	35 (79.55%)	0.51
HPV16/18 or ***FAM19A4*** positive	68	34 (77.27%)	0.5
HPV16/18 or ***MAL*** positive	72	32 (72.73%)	0.44
HPV16/18 or ***CADM1*** or ***FAM19A4*** positive	88	40 (90.91%)	0.45
HPV 16/18 or ***CADM1*** or ***MAL*** positive	92	39 (88.63%)	0.42
HPV 16/18 or ***FAM19A4*** or ***MAL*** positive	91	39 (88.63%)	0.43
HPV16/18 or ***CADM1*** or ***FAM19A4*** or ***MAL*** positive	99	42 (95.45%)	0.42
HPV16/18/52/58	77	38 (86.63%)	0.49
HPV16/18/52/58 or ***CADM1*** positive	94	42 (95.45%)	0.45
HPV16/18/52/58 or ***FAM19A4*** positive	85	40 (90.91%)	0.47
HPV16/18/52/58 or ***MAL*** positive	83	41 (93.18%)	0.49
HPV16/18/52/58 or ***CADM1*** or ***FAM19A4*** positive	96	42 (95.45%)	0.44
HPV16/18/52/58 or ***CADM1*** or ***MAL*** positive	102	42 (95.45%)	0.41
HPV16/18/52/58 or ***FAM19A4*** or ***MAL*** positive	109	42 (95.45%)	0.38
HPV16/18/52/58 or ***CADM1*** or ***FAM19A4*** or ***MAL*** positive	109	42 (95.45%)	0.38

HPV; human papillomavirus, HSIL+; High-grade squamous intraepithelial lesion or worse

**Fig 6:**
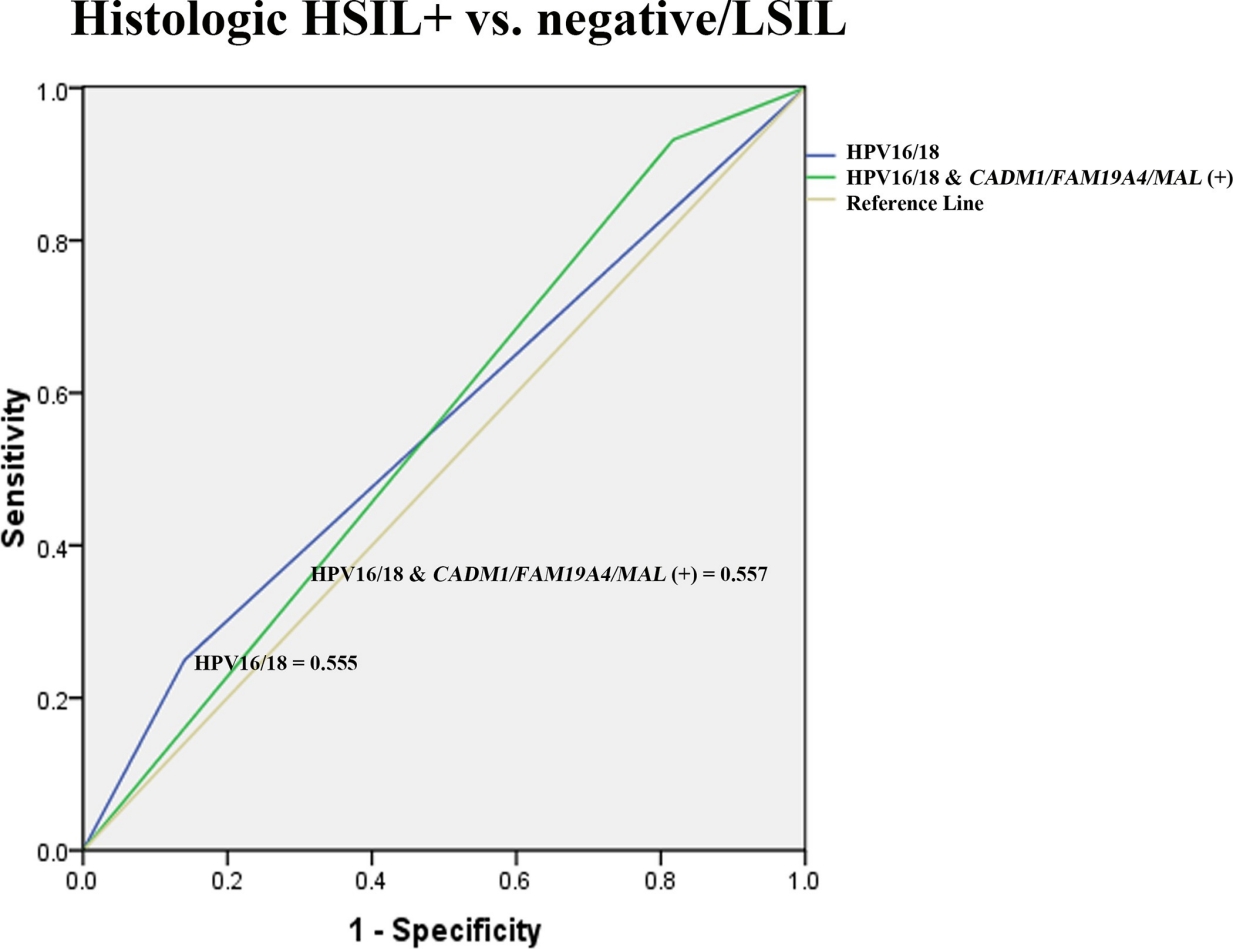
The ROC curve analysis of HPV16/18 and the combination of DNA methylation markers and HPV16/18 in differentiating histologic HSIL+ from negative/LSIL.

Interestingly, after combining positive DNA methylation status to HPV16/18 genotypes, the detection rates of histologic HSIL+ lesions were substantially increased from 25% to 79.55% for *CADM1*, 77.27% for *FAM19A4*, and 72.73% for *MAL*, respectively. The rates further increased up to 95.45% when any of these three genes was positive from methylation with comparable AUC to HPV16/18 (Fig 6). However, this suggests a possible role of genomic DNA methylation to increase sensitivity in detecting histologic HSIL+ lesions in combination with HPV tests which mostly report specific genotypes for only HPV16/18.

While combining DNA methylation markers to HPV16/18/52/58 genotypes were also increased histologic HSIL+ detection from 86.63% to 95.45% for *CADM1*, 90.91% for *FAM19A4*, and 93.18% for *MAL*, respectively.

Moreover, the multiple logistic regression analysis was used to investigate the correlation between methylation markers, HPV genotyping, and histology. High levels of *MAL* and *FAM19A4* methylation were correlated with histologic abnormalities, independent of HPV genotypes. While high *CADM1* methylation levels and the presence of HPV16/18/52/58 were independently correlated with histologic HSIL+

## Discussion

This study aimed to determine the relationship between genomic DNA methylation status and HPV genotyping for detecting the histologic HSIL+, an endpoint for cervical (pre)cancer, in hrHPV-positive women in Northern Thailand. The results showed that the cellular promoter methylation of *CADM1*, *FAM19A4*, and *MAL* was increased in the severity of cytologic abnormality, especially in cytologic HSIL comparing with NILM in HPV-positive women. Moreover, the methylation levels of these genes were also significantly higher in histologic HSIL+ than negative histology.

Using ROC analysis, the promoter methylation of *FAM19A4* was the best predictor among three investigated genes for distinguishing high-grade from non high-grade cytologic abnormalities (cytologic ASC-H+ vs. NILM/ASC-US/LSIL) in HPV-positive women. This is consistent with several studies that proposed *FAM19A4* as a marker for detecting cervical (pre)cancer [10, 19, 20, 28] in hrHPV-positive women. The POBASCAM cohort demonstrated that *FAM19A4* methylation was a potential marker for detecting cervical carcinoma and CINII/III+ lesion in hrHPV-positive women [10]. The COMETH cohort also showed a similar result of *FAM19A4* methylation in CINIII+ identification in hrHPV‐positive women aged ≥30 years with a significantly higher specificity than cytology (62.1% vs. 47.6%) and comparable sensitivity (88.3% vs. 85.5%) [19].

However, in the present study, *CADM1* methylation showed the best performance in distinguishing histologic HSIL+ from negative/LSIL while *MAL* methylation was the best predictor in differentiating abnormal from normal histology (histologic LSIL+ vs. negative).

A reduction in *CADM1* expression due to promoter methylation has been reported to be implicated in the progression of cervical lesion [7]. Overmeer and colleagues (2008) also demonstrated that the dense methylation (≥ 2 methylated regions) of *CADM1* was found at high proportion and associated with CINIII+ lesions in hrHPV-positive women [8]. The same group subsequently evaluated methylation status of *CADM1* and *MAL* genes using quantitative methylation-specific PCRs (qMSP) technique [14]. This study found that the detection rate of CINIII lesions was up to 97% and 99% for squamous cell carcinomas/adenocarcinomas in hrHPV-positive women if there was at least one gene positive for promotor methylation [14]. This is consistent with the result of the present study that combining DNA methylation to HPV16/18 substantially increased rates of histologic HSIL+ detection from 25% to 79.55% for using *CADM1* alone, 77.27% for using *FAM19A4* alone, 72.73% for using *MAL* alone and up to 95.45% for evaluating three genes in combination. Interestingly, the performance of combined *CADM1* and *MAL* methylation have been shown to be comparable to cytology or cytology combined with HPV16/18 genotyping [13]. Moreover, the methylation levels of *CADM1* and *MAL* have also been demonstrated to increase with the severity of cervical lesion in hrHPV-positive women [16]. Compared to normal cytology with histology ≤ LSIL, the methylation levels of *CADM1* and *MAL* increased 5.3 and 6.2 folds in CINII/III, and 143.5 and 454.9 folds in carcinomas, respectively [16].

Although HPV16 and HPV18 have been well recognized as the most common cause of cervical carcinogenesis, HPV58 and HPV52 are also commonly found in Asia. HPV58 was detected in 15% of squamous cell carcinoma in China [29], 16% in South Korea [30], 10% in Hong Kong [31] and 8% in Japan [32]. while HPV52 was found in 15% of squamous cell carcinoma in China [29], and 7% in Japan [32]. In the present study, a significantly higher proportion of HPV52 and HPV58 was found in histologic HSIL+ than histologic LSIL. Our previous study has also reported the high prevalence of HPV52 and HPV58 infection in Thai women with histologic HSIL+ [27]. The present study has demonstrated that using HPV16/18/52/58 genotypes, the histologic HSIL+ detection rate increased from 25% (HPV16/18) to 86.63% (HPV16/18/52/58). Moreover, combining the positive DNA methylation to HPV16/18/52/58 genotypes further increased the histologic HSIL+ detection rate to 95.45% for *CADM1* alone, 90.91% for *FAM19A4* alone, 93.18% for *MAL* alone, and 95.45% for any of these three genes.

Collectively, the combination between DNA methylation status and HPV genotype might be an alternative marker for improving the histologic HSIL+ detection in HPV-positive women. This has been recently suggested as a molecular classifier of Pap smear [33].

Recently, the co-testing of cytology and hrHPV detection has been increasingly used for cervical cancer screening [34, 35]. In this present study, there were 115 cases undergoing colposcopic examination following both cytologic abnormality and hrHPV-positivity. Forty-four cases of these were finally proven to be HSIL+ by histology (ratio = 0.382). If only cases with HPV16/18 are selected from these 115 cases for colposcopy, 33 cases of histologic HSIL+ lesions will be missed and a higher ratio of histologic HSIL+ detection (from 0.382 to 0.52) (Table 7). If cases with HPV16/18/52/58 are selected, the number of histologic HSIL+ missing will reduce from 33 to 6 cases while the detection ratio remains the same (0.49). However, most commercial test kits for hrHPV only specify HPV16 and HPV18 genotypes, not including HPV52 and HPV58. On the contrary, if we combine DNA methylation status to HPV16/18, as shown in the present study that cases with HP16/18 and high methylation level of any of 3 candidate genes (*CADM1*, *FAM19A4* and *MAL*) were selected, 42 HSIL+ lesion will be detected from colposcopies (ratio = 0.42). When considering, the 2 missing cases, one was diagnosed as cytologic ASC-H with HPV39 infection and the other was cytologic LSIL with HPV11/73 infection. This indicates that even DNA methylation appears to be useful to detect a high ratio of histologic HSIL+, cytology still cannot be definitely omitted from cervical cancer screening.

In summary, we found that DNA methylation levels correlated with cytologic and histologic grades, especially *CADM1* which significantly increased in histologic HSIL+ compared with negative histology and histologic LSIL. This supports the concept of using genomic DNA methylation as a molecular modifier in cervical cancer screening, especially in women with HPV16/18 which are generally specified by most commercial test kit. However, one of the limitations of this retrospective study is the small number of selected abnormal cytologic with hrHPV-positive samples that could only provide evidence for a proof of concept study. Future study in a larger population without case selection for methylation test would further verify the role of DNA methylation in cervical cancer screening.
